# Social and Cultural Factors Affecting Complementary and Alternative Medicine (CAM) Use during Menopause in Sydney and Bologna

**DOI:** 10.1155/2013/836234

**Published:** 2013-12-26

**Authors:** Corinne van der Sluijs, Flavia L. Lombardo, Grazia Lesi, Alan Bensoussan, Francesco Cardini

**Affiliations:** ^1^The National Institute for Complementary Medicine, University of Western Sydney Campbelltown Campus, Goldsmith Ave, Campbelltown, NSW 2560, Australia; ^2^National Center for Epidemiology, Health Surveillance and Promotion (CNESPS), National Institute of Health, Viale Regina Elena 299, 00161 Rome, Italy; ^3^Primary Care Department, Bologna Local Health Unit, Via S. Isaia 94/2, 40100 Bologna, Italy; ^4^Healthcare and Social Agency of Emilia Romagna Region, Viale Aldo Moro 21, 40127 Bologna, Italy

## Abstract

*Background*. Previous surveys found CAM use during menopause to be popular. This paper compares the results from two surveys (Sydney and Bologna) to examine factors that determine the extent and pattern of CAM use to alleviate menopausal symptoms. *Methods*. Women, aged 45–65 years, who were symptomatic when transitioning through menopause or asymptomatic but taking menopause-specific treatments, were recruited in Sydney (*n*=1,296) and Bologna (*n*=1,106) to complete the same voluntary, anonymous, and self-administered questionnaire. The results were reanalysed using stratified analyses to determine similarities and differences. *Results*. Demographics of the two cohorts differed significantly. CAM was more popular in Sydney. The most significant determinants of CAM use were the use of CAM for other conditions besides menopause and the severity of vasomotor symptoms. Occupational status was a determinant of CAM use amongst Bologna respondents only. In order to relieve symptoms, Australian and Italian women used different CAM modalities whose effectiveness was generally perceived as good. *Conclusion*. CAM use is popular amongst menopausal women from Sydney and Bologna. Differences in the patterns of CAM use seem to depend on CAM availability and on the educational level and professional status of users. The complex interaction between market, social, and cultural factors of CAM use seems to be more influential on women's choice of CAM than the available evidence of their effectiveness.

## 1. Introduction

Complementary and alternative medicine (CAM) use is popular amongst middle aged women for the alleviation of symptoms related to menopause [1–4]. The most commonly used CAM therapies include herbal products such as red clover (*Trifolium pratense*), black cohosh (*Cimicifuga racemosa*), and soy (*Glycine max*); practitioner centred modalities such as acupuncture, naturopathy, and homeopathy; and body work therapies including chiropractic and massage.

We conducted two surveys, one in Sydney (Australia) and the other in Bologna (Italy), using the same reliable and validated questionnaire to investigate the use of CAM amongst women transitioning through menopause [1, 2]. For consistency of this research, we defined CAM as any nonconventional medical practices in Australia and Italy. The term “CAM products” refers to complementary medicines approved for over the counter sale. “CAM practitioners” denoted health-care providers who may or may not be medical doctors, administering CAM treatments or recommending CAM products.

Our findings indicated that a significant number of women in both countries had used CAM treatments or products for the alleviation of symptoms and to improve quality of life and that most women had perceived these interventions to be somewhat effective [1, 2]. However, we also noted significant differences in the prevalence and pattern of CAM use. This paper investigates and proposes social and cultural drivers that may impact on and influence the uptake of CAM for the alleviation of menopausal symptoms by women in the two samples.

## 2. Methods

Detailed information of the two original surveys has been published in peer reviewed journals [1, 2]. Approvals were granted by the relevant ethics committees and government agencies. Recruitment of participants from specialist health clinics and government agencies occurred in Sydney from July 2003 until July 2004 and in Bologna from November 2004 until November 2006. Women were invited to complete the voluntary and anonymous questionnaire during a clinic visit or online if working at a government agency. Respondents were excluded if they were not aged 45–65 years, completed less than 80% of the questionnaire, were illiterate in English or Italian, or were asymptomatic and not taking treatments specifically for menopausal symptoms. The 19-item questionnaire was developed in English, then validated, and tested for reliability. The English questionnaire was translated into Italian and cross-checked for accuracy by translating it back into English. The list of CAM products and practitioners included modalities most likely to be used by women in their respective cities. The questionnaires gathered data on general demographic and health characteristics, menopausal symptoms, the use of CAM and pharmaceutical treatments during the previous 12 months, and a rating of the effectiveness of CAM treatments used to alleviate symptoms. Symptom severities were recorded on a scale from 0 (not bothered at all) to 6 (extremely bothered). Respondents were also asked where they obtained information and advice about CAM and if they had informed their doctor about their use of CAM. Sample sizes for both studies were calculated based on results from previous studies [3, 4] and were considered large enough to make meaningful comparisons between the Sydney and Bologna samples.

We reanalysed and compared the results of the studies using the chi-square test for categorical variables, the *t*-test for continuous and normally distributed variables, and the Mann-Whitney test for ordinal variables. In order to further scrutinise predictors for CAM use, a multivariate robust Poisson regression model to detect these variables was applied. Univariate analyses were undertaken to determine potential predictors, and the backward stepwise procedure was used to determine the best predictors. The final model was obtained by selecting only the significant variables, after checking that excluded variables did not affect the model. We fitted the model separately for the two samples, because of the wide differences between the characteristics of the recruited women. Statistical significance was set at 5% and all analyses were performed using Stata version 10 (Stata Corporation LP, College Station, TX, USA).

## 3. Results

A total of 2,402 eligible women (1,296 from Sydney and 1,106 from Bologna) were included in the analysis. Nearly all variables, including basic demographics, CAM, and pharmaceutical use, were significantly different between the two samples (*P* < 0.001, Table 1). However, symptom prevalence and severity reported by the two samples were similar (Table 2).

CAM use was more popular amongst women from Sydney (53.8%) than Bologna (33.5%). Although the proportion of women consulting CAM practitioners in both cities was similar (20.3% Sydney, 23.5% Bologna, *P* = 0.059), significantly more Sydney women used CAM products (48.7% Sydney, 23.6% Bologna, *P* < 0.001). The percentage of women who had not used any treatment for menopausal complaints during the previous 12 months was significantly lower in Sydney than Bologna (35.2% versus 56.2%, *P* < 0.001). A greater percentage of Sydney women used hormone therapy (HT) (*P* < 0.001) and other drugs (*P* = 0.046), also in conjunction with CAM products, compared to women from Bologna. Besides these differences, the concomitant use of CAM and other nonmenopausal drugs was common in both samples.

The differences in the use of CAM practitioners and products by the two cohorts are described in Table 3. Significantly more Sydney women, than Bolognese women, used a CAM treatment for the alleviation of menopausal symptoms during the previous 12 months (53.8% versus 33.5%), and these women were also more likely to use CAM for other conditions besides menopausal complaints (25.3% versus 15.8%; refer to Table 1). As noted in Table 3, the most commonly consulted therapists by women in Sydney were the naturopath and acupuncturist, while women from Bologna were more likely to see a herbalist or nutritionist. The most popular products were dietary soy and evening primrose oil (*Oenothera biennis*) for Sydney women and soy capsules or pills and dietary soy for women from Bologna.

Variables regarding basic demographics, current health status, pharmaceutical drug use, and menopausal symptoms were reexplored to predict CAM use.  Table 4 presents the prevalence ratios (PR) obtained from the multivariate Poisson model. The most significant determinants of CAM use amongst respondents in both studies were the use of CAM for other conditions besides menopause and the severity of vasomotor symptoms. CAM use in Sydney was associated with HT use, good or excellent health status, and sleeping difficulties, while CAM use in Bologna was more frequently associated with either being unemployed or holding a professional position, surgically induced menopause, a longer time of lapse since last menses, increased dizziness, and increased vaginal dryness. A similar association between CAM and occupational status was noted amongst women from Sydney; however these differences were not significant, possibly due to the very low proportion of unemployed women in this sample. Thus, occupational status was found to be a determinant of CAM use only amongst Bologna respondents.

## 4. Discussion

While overall CAM use was reported to be higher in Sydney than Bologna, the majority of this difference was related to the greater popularity of CAM products. Sydney women were twice as likely to use CAM products to alleviate menopausal complaints. We also noted that women from both samples often used CAM along with pharmaceutical drugs. As mentioned previously, this finding along with a lack in communication between GPs and their patients about CAM use [1, 2] should urge health care providers to be more vigilant when taking case histories so as to lessen the possibility of interactions between treatments or their misuse.

Vasomotor symptoms are generally described as the most troubling menopausal symptom for which women seek treatment [5]. Amongst respondents from both samples we found that symptomatic women who have had a previous successful encounter with CAM were more highly motivated to alleviate menopausal symptoms, using such treatments that were expected to cause fewer side effects than pharmaceuticals such as HT [6–9].

Demographic homogeneity of the Bologna cohort and heterogeneity of the Sydney cohort are supported by our data. Demographic variables indicated that the Bolognese sample was significantly more homogeneous in nature, with most women being born in Italy (98%) and speaking Italian at home, whereas a greater number of Sydney women were born overseas (73%) and/or spoke a second language. The number of women who reported being unemployed or not actively seeking employment in Bologna was significantly higher than that in Sydney, although the percentage of professionals was reportedly similar for both cities (44% Bologna and 50% Sydney). Being an unemployed Bolognese woman was significantly associated with lower CAM use. However, a significant number of women who were not employed may have been enjoying retirement. We did not ask specific questions regarding income, so we could not postulate differences in the affordability of complementary medicines or their treatments.

A poorer status of health [10, 11] and the presence of troubling symptoms [12, 13] are common reasons for the utilisation of CAM. However, because high CAM users in Sydney reported general good health, there may be additional factors that influence the uptake of CAM by women. Therefore, we postulate that a greater ease of access in purchasing CAM products and consulting with CAM practitioners in Sydney and, in the case of the analysed samples, the higher educational and professional status of respondents may all contribute somewhat to explain the differences in CAM use [14, 15]. Respondents from Sydney and Bologna were primarily recruited from governmental health services which included specialist menopause centres and family planning clinics who assist and treat women experiencing troublesome menopausal symptoms. These conventional health care services are free to the public and aim to assist those who cannot afford specialist medical care. The centres in Bologna were located in areas with a high proportion of low income families, including migrants who came mostly from the south of Italy. The Sydney menopause centres were associated with a major hospital and consulted women who resided within the local Area Health Service; consequently these women came from more diverse demographic and economic backgrounds.

Differences between Australia and Italy in governmental regulations and controls on CAM are likely to influence availability and accessibility of complementary medicines. At this point in time, Italy does not have a national registration scheme for CAM practitioners. Therefore, Bolognese women rely more on medical doctors for advice, prescriptions, and the administration of CAMs, such as acupuncture and Chinese herbal medicine. Moreover, in Bologna, only a limited number of CAM products can be purchased at supermarkets, pharmacies, or specialist herbal stores. The greater regulatory freedoms enjoyed by Australian consumers have allowed for the media marketing of CAM products and access to a wider range of CAM practitioners, encouraging the exploration of their use by consumers [16]. In Australia, a large variety of CAMs are freely available for purchase at supermarkets, pharmacies, and health food stores and over the internet. A number of CAM modalities are regulated health professions in Australia, including chiropractors, osteopaths, and more recently Chinese medicine practitioners (herbalists and acupuncturists) since July 2012. Registration will probably allow practitioners to draw greater subsidies and encourage the further integration of CAM with conventional care. There is increased medical referral of patients to CAM practitioners and greater incorporation of CAM within medical practices [17, 18].

A number of limitations to our methodologies make the extrapolation of our results to the general population of all menopausal women aged 45 to 65 difficult. The use of convenience sampling in both surveys may have overinflated the use of CAM and the reporting of menopausal symptoms, especially since many women were recruited through medical clinics. We were unable to determine an accurate response rate to our surveys due to the large number of clinics involved and because we were unable to access information regarding the numbers of eligible women working at government agencies. Furthermore, the voluntary nature of self-administered questionnaires may have selected respondents more interested in CAM, thereby further overestimating the true prevalence of CAM use.

Despite these limitations, our surveys provide relatively large data sets on the use of CAM over 12 months using the same validated and standardised questionnaire, thereby allowing us to compare two geographically and culturally diverse cities and generate an interesting insight into community trends. This comparison allowed us to identify the shared determinants of CAM use during the menopausal transition, in particular the previous use of CAM and the severity of vasomotor symptoms.

## 5. Conclusion

The emergent variations in the pattern of CAM choice between the two cities seem to depend on differences in the availability of CAM within the two countries and in the educational attainment and professional status of users. The availability of a variety of CAMs in different countries seems to be the product of local historical evolutionary factors which have shaped provision, demand, and local market structures and/or pressures. Conversely, the observed differences in educational and professional status of the two populations may have induced and allowed for different choices in response to similar symptoms of similar severity. Interestingly, the complex interaction between these market, social, and cultural factors seems to have been more influential on women's choice in the use of different CAMs than the available evidence of their effectiveness. This intriguing observation should not lessen but multiply our efforts to realise good quality research in CAM for menopausal complaints, to disseminate sound information to both health care providers and patients, and to assure equal access to the treatments of proved effectiveness.

## Figures and Tables

**Table 1:** Basic demographic characteristics, medicine use and health status of women living in Sydney and Bologna.

Variables	Sydney (*N* = 1296)		Bologna (*N* = 1106)		*P*
Age (y), mean ± SD	52.5 ± 5.08		56.0 ± 5.3		<0.001
Marital status, *n* (%)					
No partner	379 (29.7)		249 (22.7)		
With partner	899 (70.3)		849 (77.3)		<0.001
Education, *n* (%)					
Primary or secondary school	673 (52.7)		926 (83.9)		
University	603 (47.3)		178 (16.1)		<0.001
Occupation, *n* (%)					
Unemployed	75 (5.8)		454 (41.8)		
Non professional	560 (43.6)		157 (14.5)		<0.001
Professional	648 (50.5)		475 (43.7)		
Birth place, *n* (%)					
Study country	939 (73.3)		1074 (98.0)		
Other country	342 (26.7)		22 (2.0)		<0.001
Last menses, *n* (%)					
>12 months	779 (60.7)		670 (64.2)		
2–11 months	182 (14.2)		140 (13.4)		<0.05
Last month	306 (23.9)		192 (18.4)		
Surgical menopause	16 (1.2)		42 (4.0)		
Use of CAM for menopausal symptoms, *n* (%)	697 (53.8)		370 (33.5)		<0.001
Type of CAM treatment, *n* (%)					
Products	631 (48.7)		247 (23.6)		<0.001
Practitioners	263 (20.3)		255 (23.5)		0.059
Use of CAM for other conditions, *n* (%)					
No	958 (74.7)		856 (84.2)		
Yes	325 (25.3)		161 (15.8)		<0.001
Use of HT, *n* (%)					
No	872 (67.4)		933 (85.4)		
Yes	421 (32.6)		160 (14.6)		<0.001
Use of other drugs, *n* (%)					
No	518 (40.1)		481 (44.2)		
Yes	774 (59.9)		608 (55.8)		0.046
No treatment for menopausal symptoms, *n* (%)	456 (35.2)		622 (56.2)		<0.001
Current health status, *n* (%)					
Poor (1–3)	26 (2.0)		104 (9.5)		
Good (4-5)	695 (53.8)		587 (53.5)		<0.001
Excellent (6-7)	573 (44.2)		406 (37.0)		
Health status compared with 1 y ago, *n* (%)					
Worse (1–3)	72 (5.6)		188 (17.3)		
Same (4-5)	791 (61.3)		574 (52.7)		<0.001
Better (6-7)	428 (33.2)		328 (30.1)		

HT: hormone therapy; CAM: complementary and alternative medicine. Current health status ranked on a scale from 1 (poor) to 7 (excellent); health status compared with 1 year ago ranked on a scale from 1 (worse) to 7 (better).

**Table 2:** Comparison of symptom prevalence (for whole samples) and perceived severity (for CAM users).

Symptom	Prevalence			Severity*		
	Sydney (*N* = 1296)	Bologna (*N* = 1106)	*P*-value	Sydney (*N* = 697)	Bologna (*N* = 370)	*P*-value
	%	%		Mean (SD)	Mean (SD)	
Hot flush	49.4	51.0	0.453	2.5 (2.2)	2.7 (2.2)	0.260
Night sweats	47.6	45.0	0.211	2.4 (2.1)	2.4 (2.2)	0.771
Heart beats strong/quick	39.8	45.1	0.012	1.7 (1.9)	2.0 (1.8)	<0.01
Tense	64.6	64.0	0.759	2.8 (1.9)	2.8 (2.0)	0.725
Sleeping difficulties	65.5	55.7	<0.001	3.0 (2.1)	2.8 (2.1)	0.112
Panic attacks	29.1	21.4	<0.001	1.4 (1.9)	0.9 (1.5)	<0.001
Mood	53.9	54.6	0.739	2.4 (2.0)	2.4 (1.9)	0.931
Dizziness	32.0	37.6	0.006	1.4 (1.8)	1.5 (1.8)	0.331
Headache	49.4	36.4	<0.001	2.1 (1.9)	1.5 (1.8)	<0.001
Muscular pain	63.3	66.3	0.134	2.7 (2.0)	2.9 (2.1)	0.173
Crawling under the skin	23.0	40.9	<0.001	1.2 (1.8)	1.8 (2.0)	<0.001
Breathing difficulties	21.4	20.6	0.658	1.0 (1.6)	0.9 (1.6)	0.398
Menstrual irregularities	20.0	14.8	0.011	0.9 (1.7)	0.7 (1.6)	0.071
Bladder infections	11.0	10.8	0.902	0.5 (1.2)	0.5 (1.2)	0.317
Vaginal dryness	35.2	44.2	<0.001	1.7 (2.0)	2.1 (2.2)	<0.01

*Symptom severities were recorded on a scale from 0 (not bothered at all) to 6 (extremely bothered).

**Table 3:** Frequency of the most visited practitioners and the most used CAM products (of total samples) for Sydney and Bologna.

	Sydney		Bologna		*P*
	*N*	%	*N*	%	
Practitioners					
Herbalist	59	4.6	112	10.1	<0.001
Nutritionist	55	4.2	79	7.1	<0.01
Naturopath	93	7.2	29	2.6	<0.001
Acupuncturist	62	4.8	50	4.5	0.757
Chinese medicine	32	2.5	24	2.2	0.628
Homeopath	29	2.2	64	5.8	<0.001
Products					
Increased soy-based foods	329	25.4	89	8.1	<0.001
Soy and phytoestrogens (pills, capsules)	56	4.3	98	8.9	<0.001
Herbal treatments (pills, decoctions, single herbs, formulae):	289	22.3	155	14.0	<0.001
*Cimicifuga racemosa *	154	11.9	36	3.3	<0.001
*Angelica sinensis *	45	3.5	5	0.5	<0.001
Other herbal treatments (single, formulae)	154	11.9	47	4.3	<0.001
Evening primrose oil	238	18.4	n.g.		
Homeopathy products	n.g.		37	3.4	

n.g.: not gathered.

**Table 4:** Multivariate Poisson models: comparison of the predictors of CAM use by women living in Sydney and Bologna.

	Sydney			Bologna		
	PR	*P*	(95% C.I.)	PR	*P*	(95% C.I.)
Present health status	1.07	0.004	(1.02–1.11)			
Use of CAM for other conditions	1.57	0.000	(1.44–1.73)	2.45	0.000	(2.08–2.89)
HT use	1.12	0.025	(1.01–1.24)			
Hot flush severity	1.08	0.000	(1.05–1.10)	1.09	0.000	(1.04–1.15)
Sleep severity				1.06	0.005	(1.02–1.11)
Dizziness severity	1.04	0.004	(1.01–1.07)			
Vaginal dryness severity	1.03	0.031	(1.00–1.05)			
Last natural menses:						
2–11 months versus last month				1.41	0.023	(1.05–1.90)
≥12 months versus last month				1.34	0.097	(0.95–1.90)
Occupation:						
non professional versus none				1.11	0.515	(0.81–1.53)
professional versus none				1.38	0.002	(1.13–1.69)

PR: prevalence ratios. Current health status included as a continuous variable (range: 1–7); symptom severity included as continuous variable (range: 0–6).
